# The Effectiveness of Compensatory Cognitive Interventions in Mild Cognitive Impairment: A Meta-Analysis

**DOI:** 10.21203/rs.3.rs-10655912/v1

**Published:** 2026-08-12

**Authors:** Anne Milner, Tanya Horwitz, Diya Tuli, Suqing Xie, Diya Sharma, Rachel Alison De La Merced, Allison Harvey

**Affiliations:** University of California, Berkeley; University of California, Berkeley; University of California, Berkeley; University of California, Berkeley; University of California, Berkeley; University of California, Berkeley; University of California, Berkeley

**Keywords:** Mild Cognitive Impairment, memory, cognitive interventions, compensatory strategies

## Abstract

**Background:**

Cognitive interventions that incorporate compensatory strategies may be particularly effective for individuals with Mild Cognitive Impairment (MCI). This meta-analysis evaluated the efficacy of compensatory cognitive interventions for individuals with MCI. Specifically, the following were evaluated: (1) the overall efficacy across cognitive outcomes, (2) effectiveness on different cognitive domains, and (3) influence of study-level moderators.

**Method:**

Database searches were conducted, and relevant studies were screened for inclusion criteria and underwent data extraction. A meta-analysis was performed across 23 included studies, followed by subgroup moderator and sensitivity analyses.

**Results:**

The overall pooled effect size across all cognitive outcomes was *g* = 0.37, though sensitivity analyses adjusting for small-study bias suggested an effect in the small range (*g* = 0.22). Domain-specific analyses indicated significant positive effects on mental status and general cognition (*g* = 0.31), memory (*g* = 0.43), working memory and attention (*g* = 0.44), executive functioning (*g* = 0.28), and language (*g* = 0.29). Subgroup analyses revealed no overall significant differences between intervention approach, strategy type, control type, or intervention format. However, multicomponent approaches yielded significant effects for executive functioning and working memory, external strategies showed larger effects for memory and working memory/attention, and group-based formats provided greater improvements across outcomes.

**Conclusion:**

Compensatory-based interventions offer small-to-moderate benefits for individuals with MCI, particularly in the domains of memory and attention/working memory. While study-level moderators did not demonstrate significant differences, trends suggest that multicomponent approaches, using both external and internal strategies, and a group intervention format may confer some benefits.

## Background

Mild Cognitive Impairment (MCI) represents a transitional stage between age-related cognitive decline and dementia (Petersen et al., 2001). The most widely used diagnostic criteria for MCI include: 1) subjective cognitive complaints, 2) objective impairment in one or more cognitive domains, 3) preserved independence in everyday functioning, and 4) does not meet the criteria for dementia (Albert et al., 2011; Petersen et al., 1999; Winblad et al., 2004). MCI affects multiple cognitive domains, including memory, language, attention, visuospatial functioning, speed of processing, and executive functioning (Kramer et al., 2006; Lenzi et al., 2011; Winblad et al., 2004). MCI is also a significant public health concern, affecting 12–18% of adults over the age of 60 (Alzheimer’s Association, 2022), and is a risk factor in progression to dementia, with 10–15% of individuals progressing to dementia within one year (Petersen et al., 2001). Given this risk of progression, the broad impact of MCI across different cognitive domains, and the increased need for more frequent healthcare visits with age (Zhu et al., 2013), there is a clear need to develop and evaluate interventions that support cognitive functioning in individuals with MCI.

Cognitive training is a non-pharmacological approach that incorporates evidence-based strategies and skills to improve performance across cognitive domains. To date, evidence supporting cognitive interventions for individuals with MCI has been mixed. Several systematic reviews and meta-analyses have shown clear benefits (Belleville, 2008; Faucounau et al., 2010; Gomez-Soria et al., 2022; Hill et al., 2017; Jean et al., 2010; Li et al., 2011; Reijnders et al., 2013; Salzman et al., 2022), while other reviews have found little-to-no benefit (Gates et al., 2011; Huckans et al., 2013; Kurz et al., 2011; Martin et al., 2011; Stott & Spector, 2011). The lack of consensus on this topic is frequently attributed to methodological limitations, including small sample sizes, lack of appropriate control groups, and heterogeneity across interventions, which makes comparison and synthesis across studies challenging.

Cognitive training is typically categorized into two types of approaches: *restorative* and *compensatory* (Benedict, 1989; Ben-Yishay & Diller, 1993; Wilson, 1999). Restorative strategies involve enhancing or restoring cognitive abilities through repeated practice of cognitive tasks and mental exercises. In contrast, compensatory strategies are designed to teach individuals skills and strategies for performing cognitive tasks to compensate for cognitive impairments, e.g., by providing skills to change the way a patient learns, retains, or retrieves information. Compensatory strategies have also been further categorized into two distinct types of strategies: internal and external (see Table 1). External compensatory strategies involve supports such as calendars, to-do lists, and prompts in an individual’s environment that can help improve everyday functioning by providing tools to decrease demand on memory and facilitate task completion (e.g., Belleville et al., 2006; Wilson, 1999). Internal compensatory strategies are cognitive techniques that individuals can use to improve encoding of information in a meaningful way and to improve subsequent recall, and include techniques such as categorization, chunking, method of loci, and mental imagery (e.g., Belleville et al., 2006; Breuil et al., 1994; Londos et al., 2008).

Despite the distinct approaches to cognitive training outlined above, many of the interventions tested in randomized controlled trials (RCTs) are not easily categorized, as several approaches are often employed within the same intervention. These interventions are referred to as “multicomponent” and often consist of restorative and compensatory approaches, along with other types of interventions such as lifestyle modifications (exercise, diet changes, etc.) and psychotherapeutic support. Cognitive training interventions are also frequently “multi-domain,” targeting different cognitive domains such as attention, information processing, executive functioning, language, visuo-spatial functioning, and memory (Salzman et al., 2022; Sherman et al., 2020). The heterogeneity of these interventions makes it difficult to isolate the specific effects of different intervention components on improvements in patient outcomes.

However, past reviews have emphasized that cognitive interventions typically have the largest effects on memory compared to other cognitive outcomes (e.g., Simon et al., 2012). Furthermore, interventions specifically targeting memory have been associated with larger effect sizes relative to multi-domain interventions (Sherman et al., 2017, 2020). A recent meta-analysis also emphasized the efficacy of compensatory approaches to improve cognition (Sherman et al., 2020). Compensatory strategies may be particularly effective for individuals experiencing MCI because such strategies require deep processing of the information to be remembered (Chi & Wylie, 2014; Menekse et al., 2013), potentially enhancing encoding and subsequent recall more than alternative interventions. These compensatory strategies may be underpinned by constructive learning behavior, which involves actively engaging with the material by constructing new inferences, ideas, or connections, processes which have been shown to improve learning outcomes (Chi et al., 2001). This is consistent with evidence demonstrating that strategies incorporating constructive learning are more effective than non-constructive strategies such as repetition and practicing recall of information (i.e., restorative-based approaches) (Zieve et al., 2019). Furthermore, Milner et al. (2026) also found evidence that constructive strategies may be more effective than non-constructive buffering against declines in memory recall in cognitive impairment.

While recent meta-analyses have demonstrated general benefits of cognitive training on global cognition (e.g., Gomez-Soria, 2022; Salzman et al., 2022; Sherman et al., 2020), few studies have focused specifically on compensatory approaches in samples that use rigorous control groups. Given that compensatory approaches appear to be particularly effective for individuals with MCI, there is a need for a greater focus on the most recent, rigorously designed studies testing compensatory cognitive interventions. Therefore, the current study aims to provide an updated meta-analysis of the literature examining the effects of compensatory cognitive interventions exclusively in controlled studies in which patients are diagnosed with MCI according to published criteria and objective outcomes. Furthermore, this study uses a multi-level meta-analysis approach, which allows for retention of effect sizes for all outcomes within a study (i.e., not averaging or creating a composite score), significantly strengthening the validity and precision of the results.

Beyond the specific cognitive intervention components themselves, there are several methodological characteristics of clinical trials that may also impact the magnitude of the effects. In particular, the type of control group that is used is an important consideration for meta-analyses, as active controls account for non-intervention effects (e.g., social engagement, time spent) while inactive controls do not. Typically, meta-analyses find that effect sizes are greater for studies with inactive control groups relative to active (Cuijpers et al., 2024). Furthermore, whether the intervention is in group or individual format may influence effects. While individual interventions may provide some benefit as they are often tailored to each patient’s needs, group interventions may also provide some benefit through social engagement and support, which is particularly important for individuals with MCI (Leung et al., 2015). Therefore, it’s important to evaluate if these study-level characteristics impact the effects.

Aim 1 is to evaluate the overall efficacy of interventions incorporating compensatory approaches on cognitive outcomes in individuals experiencing MCI. Aim 2 is to determine whether treatment effects of compensatory interventions vary across cognitive domains of the outcome measure (e.g., general cognition, memory, working memory and attention, speed of information processing, language, visual-spatial ability, executive functioning). Aim 3 is to test the following study-level moderators on treatment effects: a) Intervention type – primarily compensatory vs. multicomponent, b) Compensatory strategy – internal vs. external, c) Control group type – active vs. inactive, d) Intervention format – individual vs. group.

## Method

### Study Selection and Analysis

We followed the reporting guidelines outlined in the Preferred Reporting Items for Systematic Reviews and Meta-Analyses (PRISMA) (Page et al., 2021) and registered the protocol with PROSPERO (CRD420251183007) on November 23^rd^, 2025.

### Eligibility criteria

The inclusion criteria for studies were as follows: (1) the study population was diagnosed with Mild Cognitive Impairment (MCI) based on established criteria (Petersen et al., 1999), (2) the tested intervention included a compensatory component; see Table 1 for a definition of possible compensatory components, (3) the study compared an intervention group with MCI to an active and/or inactive control group with MCI, (4) the study included an objective, neuropsychological outcome that assessed a cognitive domain (e.g., memory, attention), and (5) the study reported sufficient descriptive statistics (e.g., means/SDs, effect sizes, or tests statistics) to allow for quantitative synthesis.

### Search Strategy

Our search criteria were intended to identify cognitive interventions that included a compensatory component in a sample diagnosed with MCI. Studies were identified by searching the following databases: Embase, Ovid MEDLINE, PsycINFO, PubMed, and Web of Science. The search combined three main categories of keywords using the AND and OR operators: (1) MCI terms (e.g., “Mild Cognitive Impairment,” “MCI”); (2) Cognitive/compensatory terms: “memory,” “compensat*,” “external aids”; (3) Intervention terms (e.g., “interven*,” “rehab*,” “training”). The following is an example search strategy for PubMed: (“Mild Cognitive Impairment” OR “MCI”) AND (“memory” OR “compensat*” OR “cogniti*” OR “visual imagery” OR “mental imagery” OR “method of loci” OR “mind mapping” OR “categorization” OR “chunking” OR “cueing” OR “memory aids” OR “external aids”) AND (“interven*” OR “strateg*” OR “rehab*” OR “treatment” OR “stimulation” OR “training”).

Papers were also restricted to human studies, English-language publications, and clinical trials. The date of the last search was March 2026. Citations from each of these databases were uploaded to Covidence, which was used for managing the review process for the meta-analysis. Specifically, Covidence was used for detecting duplicate studies across searched databases, as well as for conducting title and abstract screening, full-text review, data extraction, and risk of bias assessment.

### Screening

All authors served as trained reviewers who independently screened papers within Covidence. During the initial screening phase, the title and abstract of each study were screened by two independent reviewers. In the second phase, two independent reviewers evaluated the full text of each paper to establish whether it met the eligibility criteria. Studies that met the eligibility criteria were advanced to the data extraction phase. The first author resolved conflicts between reviewers through consensus discussions.

### Data extraction

Two reviewers extracted data and categorized outcomes from the included studies, and the first author reached a final consensus that was used in the final analyses. Data were extracted only for objective neuropsychological outcomes and were categorized into one of the following cognitive domains: mental status and general cognition, memory, working memory and attention, executive function, language, speed of information processing, or visual-spatial ability. Means and standard deviations across measures were extracted for the post-treatment time point. If data were not collected at post-treatment, the time point closest to post-treatment was used. For subgroup analyses, study characteristics were categorized across four study-level moderators (a) intervention type (primarily compensatory vs. multi-component), (b) strategy type (internal vs. external), (c) control group type (active vs. inactive), and (d) intervention format (group vs. individual). For intervention type studies where at least half of the included components were compensatory were classed as primarily compensatory, whereas studies in which at least one but fewer than half of the components were compensatory were classed as multicomponent. For strategy type, studies were classified as internal compensatory strategies if they included mnemonic strategies, organization, categorization, mental/visual imagery, method of loci, and face-name associations, whereas they were classed as external compensatory strategies if they included the use of external aids or cues (e.g., calendars, notes, and prompts). For control group type, studies were classed as active if they included an active treatment condition as a control (e.g., treatment-as-usual, inferior treatment) or inactive (e.g., psychoeducation, waitlist control). For intervention format, studies where patients completed treatment alone were classed as individual and studies where patients attended sessions with other patients were classed as group.

### Risk of bias assessments

The original Cochrane Risk of Bias Tool (RoB 1.0; Higgins et al., 2011) was used to assess randomized controlled trials (RCTs) as either at high, unclear, or low risk of bias across seven domains: (1) sequence generation, (2) allocation concealment, (3) blinding of participants and personnel for all outcomes, (4) blinding of outcome assessors for all outcomes, (5) incomplete outcome data for all outcomes, (6) selective outcome reporting, and (7) other sources of bias. There were two non-randomized controlled trials (Moro et al., 2012; Mraz et al., 2023) that were assessed with the Risk of Bias in Non-randomized Studies (Sterne et al., 2016). The ROBINS-I comprises seven domains that are rated as either high, moderate, or low risk: (1) bias due to confounding, (2) bias in selection of participants into the study, (3) bias in classification of interventions, (4) bias due to deviations from intended interventions, (5) bias due to missing data, (6) bias in measurement of outcomes, and (7) bias in selection of the reported result.

### Analysis

All analyses were performed in R (Core Team, 2021). Standardized mean differences (SMDs) at post-treatment were used, following methodological recommendations that using change scores can result in biased outcomes when conducting a meta-analysis on RCTs (Chandler et al., 2019; Cuijpers et al., 2017). Effect sizes were calculated using the *esc* package (Lüdecke et al., 2019), where differences in means between the intervention and control groups at post-treatment were calculated as Hedges’ *g* (intervention minus control). The 95% confidence interval (CI) was also calculated for each effect. While higher scores across most outcome measures indicate better scores, the directionality of some outcome measures was reversed (e.g., ADAS-cog, CDR-SB, TMT). These measures were multiplied by −1. Thus, positive SMDs reflect a greater improvement in outcome for the intervention group than for the control group across all study outcomes.

One study (Park et al., 2019) only reported change from baseline scores and standard deviations, and the correlations between the baseline and post-treatment measures were unknown. As recommended in the Cochrane Handbook for Systematic Reviews and Interventions (Chandler et al., 2019), the baseline SD was substituted for the post-treatment SD when calculating effect sizes for this study, with the assumption that variability in outcomes was not influenced by the intervention. Jeong et al. (2016) included two active interventions: an in-person group-based intervention and a home-based intervention consisting of homework exercises. Only values pertaining to the group-based intervention were retained when calculating effect sizes, as this intervention included primarily compensatory strategies, whereas the home-based intervention did not. There was only one measure of visual-spatial ability across studies (Corsi Spatial Sequence Task; CSST). This measure was collapsed into the “working memory and attention” domain, as it also serves as a measure of spatial working memory. Two studies (Belleville et al., 2018; Olchik et al., 2013) included both an active and inactive control group. For these studies, the active control was selected for the primary analysis to provide a more conservative estimate. However, effects relative to both the active and the inactive control group were included for the sub-group analysis examining control group type as a moderator.

Multi-level random-effects meta-analyses were conducted using the *metafor* package (Viechtbauer, 2010). Models were estimated using restricted maximum-likelihood estimation (REML). For Aim 1, the overall treatment effect of compensatory interventions across all cognitive outcomes was evaluated using a multi-level model to account for multiple observations nested within each study. Level 1 accounted for the participant-level sampling error, Level 2 accounted for within-study variation (i.e., multiple outcomes reported within the same study), and Level 3 accounted for between-study variation. This was an intercept-only model, which did not include any predictors but was used to calculate an overall average effect size across all outcomes. Aim 2 tested whether treatment effects differed across cognitive domain outcomes as well as the significance of the effect associated with each type of outcome. A similar multi-level model was run, except that Aim 2 included cognitive domain of the outcome as a moderator. One version of the model retained the global intercept to provide an omnibus test for cognitive domain as a moderator. A second version of the model omitting the global intercept was also run to test whether the treatment effect associated with each cognitive domain was statistically different from 0. For Aim 3, similar multi-level models were used to examine whether treatment effects were moderated by four study-level characteristics: (a) intervention type (compensatory vs. multicomponent), (b) strategy type (external vs. internal), (c) control group type (active vs. inactive), and (d) intervention format (individual vs. group). To determine whether the impact of these study-level characteristics also depended on the cognitive domain of the outcome, a series of additional models was run. For each characteristic, a standard interaction model including both the characteristic and cognitive domain as predictors was tested. One version of the models retained the global intercept to provide omnibus tests of the interactions. A second version of the models omitting the global intercepts was also run to determine whether each of the pooled treatment effects associated with each of the characteristic x cognitive domain combinations was significantly different from 0.

## Results

Our search terms provided 2398 records after duplicates were removed. A total of 360 studies remained after title and abstract screening, and 23 studies remained after full-text review. The main reasons for exclusion were study samples not meeting established MCI criteria and tested interventions not including a compensatory component. See Figure 1 for the selection process and all reasons for exclusion. Effect sizes were calculated as Hedges’ *g* and can be interpreted as follows: very small (0.00–0.19), small (0.20–0.49), moderate (0.50–0.79), or large effect (≥ 0.80) (Cohen, 2013).

### Characteristics of included studies

See Table 2 for details of the 23 studies included in the meta-analysis, including study design, sample size, descriptives (age and mental status), intervention characteristics (format, length, components), and outcome measures. There were 21 RCTs and 2 non-randomized controlled studies. The majority of included studies tested primarily compensatory interventions (*n* = 17, 73.9%), and the remaining studies tested multicomponent interventions (*n* = 6, 26.1%). Studies primarily employed internal compensatory strategies (*n* = 15, 65.2%); only one study included purely external compensatory strategies (*n* = 1, 4.3%); and several studies employed both internal and external strategies (*n* = 7, 30.4%). Different studies also used different intervention formats, including individual sessions (*n* = 14, 60.9%), group sessions (*n* = 8, 34.8%), and both individual and group sessions (*n* = 1, 4.3%). Eleven studies had an inactive control group (47.8%), ten had an active control group (43.5%), and two had both an inactive and active control (8.7%). Studies included outcome measures from several different cognitive domains (which were not mutually exclusive within-study): mental status and general cognition (*n* = 13, 56.5%), memory (*n* = 20, 87.0%), working memory and attention (*n* = 10, 43.5%), executive function (*n* = 9, 39.1%), language (*n* = 5, 21.7%), and speed of information processing (*n* = 7, 30.4%).

### Risk of bias assessments

RCTs were assessed for risk of bias using the RoB 1.0 across seven different domains. See Figure 2 for a summary of risk of bias across the 21 RCT studies. All studies were rated as high risk for blinding of participants and personnel because participants and personnel could not be blinded, which is often unavoidable in behavioral intervention studies. Most studies were rated as low risk for sequence generation (66.7%), allocation concealment (57.1%), blinding of outcome assessors (61.9%), incomplete outcome data (71.43%), and other sources of bias (61.9%). Most studies had unclear risk for selective outcome reporting (61.9%) because most studies were not pre-registered.

The two non-randomized controlled studies (Moro et al., 2012; Mraz et al., 2023) were assessed with the ROBINS-I across seven risk domains: bias due to confounding (1 high, 1 moderate), bias in selection of participants into the study (2 low), bias in classification of interventions (2 low), bias due to deviations from intended interventions (2 moderate), bias due to missing data (2 low), bias in measurement of outcomes (1 moderate, 1 low), and bias in selection of reported result (1 moderate, 1 low). Each risk of bias domain was included as a moderator in a separate model described below (see sensitivity analyses). The lack of participant/personnel blinding domain was not included as a moderator, as all studies were rated as high risk.

### Aim 1: Overall treatment effect of compensatory cognitive interventions

The overall pooled Hedges’ *g* (across all cognitive outcomes) was 0.37 (95% CI [0.23, 0.51], *SE* = 0.07, *t*(154) = 5.22, *p* < 0.001), indicating a moderate and positive overall effect of compensatory cognitive interventions. See Figure 3 for a forest plot of these effects; note that while the full multi-level model analyzed 155 effect sizes, the plot depicts study-level composites for the 23 included studies for visual clarity. Forest plots of the 155 effect sizes are reported by cognitive domain of the outcome in Supplement Figures S1–S6.

Total heterogeneity was moderate and statistically significant (*I*^*2*^ = 59.88%, *Q*(154) = 315.64, *p* < 0.001). Of the total variation, 50.28% was attributable to between-study heterogeneity (Level 3), 9.60% to within-study heterogeneity (Level 2), and the remaining 40.13% to sampling error. The three-level model provided a significantly better fit compared to a two-level model with level 3 heterogeneity constrained to 0 (χ^2^_1_ = 41.44, *p* < 0.001).

#### Sensitivity Analyses

To evaluate whether there were any influential studies, Cook’s distance was calculated for each study. Based on the recommended threshold of 4/*n* (Bollen & Jackman, 1985), two studies—Fonte et al. (2019) and Herrera et al. (2012)—were identified as being potentially influential because their Cook’s distance values (D = 0.18 and D = 0.23, respectively) exceeded this threshold (0.17 for the current meta-analysis). Sensitivity analyses were conducted excluding these two studies from the multi-level model. The point estimate of the pooled effect after exclusion was significant (Hedges’ *g* = 0.31, 95% CI [0.19, 0.42], *p* < 0.001) and was similar to the point estimate prior to exclusion.

Visual inspection of a funnel plot revealed patterns of asymmetry that suggested small-study effects, whereby smaller studies are typically associated with larger effect sizes, which can be the result of publication bias or other sources of bias. This asymmetry was further evaluated using a multi-level model with standard error as a predictor. Larger standard errors were associated with larger effect sizes (*b* = 2.62, *SE* = 0.51, *p* < 0.001), indicating that less precise studies reported larger effect sizes. However, these findings should be interpreted with caution because publication bias diagnostics are not yet well established for multi-level meta-analyses. Given the evidence of small-sample effects, sensitivity analyses excluding individual effect sizes with a high standard error (greater than 0.40 based on visual inspection of the funnel plot) were conducted. Following exclusion, 44 of the 155 effect sizes were excluded based on this threshold; there was still a significant positive pooled effect estimate (*g* = 0.22, 95% CI [0.12, 0.33], *p* < 0.001), although the effect size was substantially smaller than when including all studies (*g* = 0.37).

Sensitivity analyses were also conducted with the risk of bias rating for each domain, as determined by the ROBINS-I or RoB 1.0, included as a moderator in separate models. Sequence generation, allocation concealment, blinding of outcome assessors, incomplete outcome data, and selective outcome reporting risk ratings did not significantly moderate outcomes. However, the other sources of bias domain significantly moderated outcomes (*F*(2, 152) = 10.24, *p* < 0.001), such that the studies rated as high risk for other sources of bias (i.e., baseline imbalances, baseline differences, etc.) had larger effects than those that were rated as low risk (*b* = −0.53, 95% CI [−0.76, −0.30], p < 0.001). There was no significant difference between unclear and high-risk studies (*b* = −0.28, 95% CI [−0.74, 0.18], *p* = 0.23). These results indicate that other sources of bias may have affected the magnitude of the effects. The primary model was re-run restricted only to studies that were rated as low-risk for other sources of bias. The pooled effect size remained significant (*g* = 0.21, 95% CI [0.10, 0.31], *p* < 0.001), demonstrating that while high-risk studies inflated the observed effect, the overall finding remains robust.

### Aim 2: Treatment effects by domain of cognitive outcome

To evaluate whether overall treatment effects differed by cognitive domain of the outcome, a multi-level meta-analysis was conducted with cognitive domain type as a moderator. The overall pooled effect did not differ significantly between cognitive domains (*F*(5, 149) = 1.53, *p* = 0.18), suggesting that outcomes across domains were comparable. As a descriptive analysis, a multi-level model omitting the global intercept was conducted to assess whether the effect size for each cognitive domain outcome was significantly different from zero. There was a statistically significant effect of the interventions on mental status and general cognition (*g* = 0.31, 95% CI [0.13, 0.50], *p* < 0.01), memory (*g* = 0.43, 95% CI [0.27, 0.58], *p* < 0.001), working memory and attention (*g* = 0.44, 95% CI [0.25, 0.62], *p* < 0.001), executive functioning (*g* = 0.28, 95% CI [0.05, 0.51], *p* = 0.02), and language (*g* = 0.29, 95% CI [0.06, 0.52], *p* = 0.01). Intervention effects on the speed of information processing were not significant (*g* = 0.21, 95% CI [−0.04, 0.45], *p* = 0.10). See Figure 4 for a forest plot of the estimated pooled effects by cognitive domain.

### Aim 3: Sub-group analyses

In our pre-registered protocol, we planned to conduct sub-group analyses to examine potential moderation effects across several variables: MCI subtype (amnestic, non-amnestic), compensatory strategy type (internal, external), control group type (active, inactive control), and intervention format (individual, group, both). The majority of the included studies examined individuals with amnestic MCI (*n* = 14), and the remaining studies did not specify whether their samples included amnestic or non-amnestic individuals. Therefore, the planned sub-group analyses for MCI subtype were not conducted. Furthermore, only a single study included a purely external compensatory approach (Greenaway et al., 2013). Therefore, the pre-registered analysis of compensatory strategies was adapted to instead compare studies that included only internal strategies (*n* = 15) to interventions including external strategies (*n* = 1 external only; *n* = 7 internal and external). Only one study (Schmitter-Edgecombe & Dyck, 2014) included both a group and individual format. Thus, this study was excluded from the intervention format subgroup analyses. Additionally, a post-hoc exploratory analysis was conducted to test the effects of intervention type (primarily compensatory vs. multicomponent). The sub-group analyses involving intervention type, compensatory strategy approach, control group type, and intervention format are reported below.

#### Aim 3a: Treatment effects by intervention approach (compensatory only versus multicomponent)

To evaluate whether overall treatment effects differed by the intervention approach across all cognitive outcomes combined, a multi-level meta-analysis was conducted with intervention type as a moderator. The overall pooled effect did not differ significantly between primarily compensatory and multicomponent interventions (*b* = 0.04, 95% CI [−0.28, 0.36], *p* = 0.81), suggesting that effect sizes for compensatory (*g* = 0.37) and multicomponent interventions (*g* = 0.40) were comparable. To determine whether the difference in treatment effects between the two intervention types varied by cognitive domain of the outcome, another similar multi-level interaction model with intervention type and cognitive domain as moderators was conducted.

The omnibus moderation effect was not statistically significant (*F*(4, 144) = 1.36, *p* = 0.25; df1 reflects the absence of language outcome for multicomponent trials). As a descriptive analysis, a multi-level model omitting the global intercept was run to assess whether the effect sizes for each level of the intervention variable were significantly different from 0 for the various cognitive domain outcomes. For the primarily compensatory intervention, positive treatment effects were observed across four domains: mental status and general cognition (*g* = 0.33, 95% CI [0.09, 0.57]*, p* < 0.01), memory (*g* = 0.44, 95% CI [0.24, 0.63], *p* < 0.001), working memory and attention (*g* = 0.37, 95% CI [0.14, 0.59], *p* < 0.01), and language (*g* = 0.27, 95% CI [0.02, 0.52], *p* = 0.03). Compensatory interventions did not have a significant treatment effect on executive functioning or speed of information processing (*p’s* > 0.10). For multicomponent interventions, positive treatment effects were observed across three domains: memory (*g* = 0.43, 95% CI [0.12, 0.74], *p* < 0.01), working memory and attention (*g* = 0.67, 95% CI [0.29, 1.05]*, p* < 0.001), and executive functioning (*g* = 0.47, 95% CI [0.04, 0.91], *p* = 0.03). Additionally, there were positive treatment effects that approached significance on mental status and general cognition (*g* = 0.32, 95% CI [−0.01, 0.65], *p* = 0.06) and on speed of information processing (*g* = 0.30, 95% CI [−0.05, 0.65], *p* = 0.09). None of the studies with a multicomponent intervention included language as an outcome. See Figure 5 for a forest plot of the estimated pooled effects for each intervention type (compensatory versus multicomponent) x cognitive domain combination.

#### Aim 3b: Treatment effects by compensatory strategy (internal versus external)

To evaluate whether overall treatment effects differed by the compensatory strategy across all cognitive outcomes combined, a multi-level meta-analysis was conducted with strategy type as a moderator. The overall pooled effect did not differ significantly between internal and external strategies (*b* = −0.07, 95% CI [−0.38, 0.23], *p* = 0.63), suggesting that effect sizes for internal (*g* = 0.35) and external strategies (*g* = 0.42) were comparable. To determine whether the difference in treatment effects between these two intervention types depended on the cognitive domain of the outcome, another similar multi-level model with strategy type and cognitive domain as moderators was conducted. The omnibus moderation effect was not statistically significant (*F*(5, 143) = 0.99, *p* = 0.43). As a descriptive analysis, a multi-level model omitting the global intercept was conducted to assess whether the effect sizes for each strategy type (internal vs. external) were statistically different from zero for each cognitive domain outcome. For the interventions including only internal strategies, positive treatment effects were observed across four domains: mental status and general cognition (*g* = 0.35, 95% CI [0.13, 0.58], *p* < 0.01), memory (*g* = 0.36, 95% CI [0.17, 0.55], *p* < 0.001), working memory and attention (*g* = 0.41, 95% CI [0.19, 0.64], *p* < 0.001), and executive functioning (*g* = 0.32, 95% CI [0.01, 0.62], *p* = 0.04). There was a positive treatment effect that approached significance on language (*g* = 0.25, 95% CI [−0.03, 0.53], *p* = 0.07). There was no significant treatment effect on speed of information processing (*p* = 0.19). For interventions that included external strategies, positive treatment effects were observed across two domains: memory (*g* = 0.60, 95% CI [0.32, 0.87], *p* < 0.001) and working memory and attention (*g* = 0.53, 95% CI [0.19, 0.87], *p* < 0.01). There was a positive treatment effect that approached significance on language (*g* = 0.40, 95% CI [−0.03, 0.83], *p* = 0.07). There was no significant treatment effect on executive functioning or speed of information processing (*p’s* > 0.10). See Figure 6 for a forest plot of the estimated pooled effects by compensatory strategy type (internal versus external) and for each cognitive domain.

#### Aim 3c: Treatment effects by control group type (active versus inactive)

To evaluate whether overall treatment effects differed by control group type across all cognitive outcomes, a multi-level meta-analysis was conducted with control group type as a moderator. The overall pooled effect between active and inactive control studies was not significant (*b* = −0.13, 95% CI [−0.33, 0.07], *p* = 0.19), suggesting that effect sizes for active (*g* = 0.44) and inactive control groups (*g* = 0.31) were comparable. To determine whether the difference in treatment effects between studies with these two control group types depended on the cognitive domain of the outcome, another similar multi-level model with control group type and cognitive domain as moderators was conducted. The omnibus moderation effect was not statistically significant (*F*(5, 153) = 1.87, *p* = 0.10). A multi-level model omitting the global intercept was conducted to assess whether the effect sizes for studies with each control group type were statistically different from zero for each cognitive domain outcome. For the active control group studies, positive treatment effects were observed across four domains: mental status and general cognition (*g* = 0.46, 95% CI [0.20, 0.72], *p* < 0.001), memory (*g* = 0.47, 95% CI [0.30, 0.65], *p* < 0.001), working memory and attention (*g* = 0.57, 95% CI [0.30, 0.85], *p* < 0.001), and speed of information processing (*g* = 0.31, 95% CI [0.02, 0.61], *p* = 0.03). There was not a significant pooled treatment effect for language in the active control group studies (*p* = 0.47). For the inactive control group studies, positive treatment effects were observed across three domains: memory (*g* = 0.36, 95% CI [0.19, 0.53], *p* < 0.001), working memory and attention (*g* = 0.34, 95% CI [0.13, 0.55], *p* < 0.01), and language (*g* = 0.32, 95% CI [0.07, 0.56], *p* = 0.01). There were no significant treatment effects on mental status and general cognition (*g* = 0.16, 95% CI [−0.06, 0.38], *p* = 0.15), executive functioning (*g* = 0.01, 95% CI [−0.29, 0.31], *p* = 0.95), or speed of information processing (*g* = 0.08, 95% CI [−0.31, 0.46], *p* = 0.70). See Figure 7 for a forest plot of the estimated pooled effects by control group type (inactive versus active) and for each cognitive domain.

#### Aim 3d: Treatment effects by intervention format (individual versus group)

To evaluate whether overall treatment effects differed by intervention format type across all cognitive outcomes combined, a multi-level meta-analysis was conducted with intervention format type as a moderator. The difference in overall pooled effect between individual and group intervention formats was not significant (*b* = −0.19, 95% CI [−0.50, 0.13], *p* = 0.24), suggesting that effect sizes for individual (*g* = 0.31) and group intervention formats (*g* = 0.50) were comparable despite falling in the moderate range for group interventions and small range for individual interventions. To determine whether treatment effect differences between these two intervention formats depended on the cognitive domain of the outcome, another similar multi-level model with intervention format and cognitive domain as moderators was conducted. The omnibus moderation effect approached statistical significance (*F*(5, 139) = 1.98, *p* = 0.09). A multi-level model omitting the global intercept was run to assess whether the effect sizes for individual and group intervention types were statistically different from zero for each cognitive domain outcome. For the individual intervention format, positive treatment effects were observed across three domains: memory (*g* = 0.37, 95% CI [0.17, 0.57], *p* < 0.001), working memory and attention (*g* = 0.45, 95% CI [0.22, 0.67], *p* < 0.001), and language (*g* = 0.33, 95% CI [0.06, 0.62], *p* = 0.02). There was no significant treatment effect on mental status and general cognition, executive functioning, or speed of information processing (*p’s* > 0.10). For the group intervention format, positive treatment effects were observed across three domains: mental status and general cognition (*g* = 0.58, 95% CI [0.26, 0.91], *p* < 0.001), memory (*g* = 0.57, 95% CI [0.30, 0.84], *p* < 0.001), and executive functioning (*g* = 0.46, 95% CI [0.04, 0.89], *p* = 0.03). See Figure 8 for a forest plot of the estimated pooled effects by intervention format type (individual vs. group) and for each cognitive domain.

## Discussion

The primary goal of this meta-analysis was to examine the impact of cognitive interventions that include compensatory strategies on outcomes in individuals with MCI and to examine potential moderators of these effects. Specifically, the first aim evaluated the overall impact of these interventions across all cognitive outcomes. There was a small-to-moderate effect size (*g* = 0.37) across outcomes. This finding suggests that, in general, individuals with MCI who are taught compensatory strategies show improvements in cognition.

The second aim evaluated whether treatment effects were moderated by the cognitive domain of the outcome measure (i.e., general cognition and mental status, memory, working memory and attention, language, and speed of processing). Although effect sizes did not differ significantly between cognitive domains, the largest improvements were observed in memory (*g* = 0.43) and working memory/attention (*g* = 0.44). There were also small effects on mental status and general cognition (*g* = 0.31), executive functioning (*g* = 0.28), and language outcomes (*g* = 0.29), but no significant effect of the interventions on speed of processing (*g* = 0.21). These findings indicate that cognitive compensatory interventions provide benefits across most cognitive outcomes for individuals with MCI. Specifically, they yield the greatest efficacy for memory and working memory/attention outcomes, but they appear not to be effective for speed of processing outcomes. It’s plausible that compensatory strategies, which are designed to reduce demands on limited cognitive resources (Clare & Woods, 2004) rather than directly remediate underlying cognition, are unlikely to result in improvements for speed of information processing. This interpretation is consistent with evidence suggesting that restorative cognitive interventions, which employ repetitive, task-specific training to enhance or restore cognitive function, can have moderate effects on speed of information processing in samples without MCI (Lampit et al., 2019). Unlike compensatory approaches, restorative interventions target underlying cognitive mechanisms via repeated practice and more direct focus on processing speed, attention, memory, and executive function (Hardy & Scanlon, 2009; Westerberg et al., 2007; Zhang et al., 2019). A valuable domain for future research is to determine if restorative approaches may be better suited to address the cognitive domains for which compensatory interventions failed to yield significant effects in patients with MCI.

The third aim was to evaluate whether treatment effects differed by the following subgroups: a) intervention type, b) compensatory strategy, c) control group type, and d) intervention format. Aim 3a evaluated the type of intervention approach that was used by testing intervention approach (primarily compensatory vs. multicomponent with at least one compensatory component) as a moderator. There were no significant differences between the two intervention approaches when collapsing across outcomes, and the results of the omnibus interaction model testing both intervention and cognitive domain as moderators were not significant. The lack of a moderation effect for the pooled results indicates that neither intervention type is superior to the other when evaluating cognitive outcomes overall. From a clinical perspective, this indicates there may be some flexibility in the type of approach used based on resources, patient preference, and clinical expertise without influencing the efficacy of the intervention. However, when examining individual cognitive domains, distinct underlying processes may dictate which intervention types provide the greatest benefit.

The two intervention types were comparable for memory (*g* = 0.43 and 0.44 for multicomponent and compensatory interventions, respectively) and for mental status and general cognition (*g* = 0.32 and 0.33, respectively). However, while the effect of multicomponent interventions was significant for executive functioning (*g* = 0.47) and approached significance for speed of information processing (*g* = 0.30), there was no significant effect for compensatory interventions on executive functioning (*g* = 0.19) or on speed of information processing (*g* = 0.02). Multicomponent interventions also showed larger effects than compensatory interventions on working memory/attention (*g* = 0.67 vs. 0.37, respectively), although both effects were significant. These results suggest that while purely compensatory approaches are most beneficial for mental status/general cognition and memory, multicomponent interventions may target a broader range of cognitive abilities, including working memory, executive function, and speed of information processing. This may be because most multicomponent interventions employ a combination of restorative and compensatory approaches, in which restorative interventions primarily target domains such as processing speed, attention, and executive function (Hardy & Scanlon, 2009; Westerberg et al., 2007; Zhang et al., 2019). This distinction has important clinical implications for designing interventions. Patients with amnestic MCI, which is primarily characterized by impairments in memory, may benefit most from compensatory interventions, while non-amnesic MCI can result in impairments in attention/concentration, information processing, language, and executive function (Luck et al., 2010; Petersen et al., 2009), which may be better targeted by restorative approaches. Unfortunately, the included studies did not allow for the analysis of MCI subtype, which will be important to establish in future work.

Aim 3b was to evaluate whether treatment effects differed by the type of compensatory strategy employed, comparing internal strategies (e.g., mental imagery, mnemonics) to external strategies (e.g., calendars, cues, and prompts). Collapsing across cognitive outcomes, there was no significant difference in treatment effects between internal and external strategies (*g* = 0.35 for internal, *g* = 0.42 for external). However, when examining the effects on outcomes from different cognitive domains, there was a significant effect of internal (*g* = 0.35) but not external strategies (*g* = 0.20) on mental status and general cognition. Treatment effects were generally larger for external compared to internal approach across the following cognitive domains: memory (*g* = 0.60 vs. 0.36), working memory and attention (*g* = 0.53 vs. 0.41), and language (*g* = 0.40 vs. 0.25). This indicates that external strategies may be effective at targeting more cognitive domains. However, more research needs to be conducted on interventions that evaluate each of these strategy types in isolation and specifically in individuals experiencing MCI.

Aim 3c was to evaluate whether treatment effects differed depending on the type of control group used, comparing inactive (did not receive any treatment) to active (received active treatment, e.g., treatment-as-usual, alternative intervention) control groups. Overall, effect sizes were not significantly different for studies with active (*g* = 0.44) compared to inactive control groups (*g* = 0.31). However, the point estimates of effect sizes were larger for studies including an active control than for those with an inactive for mental status and cognition (*g* = 0.48 vs. 0.15), memory (*g* = 0.49 vs. 0.34), working memory and attention (*g* = 0.59 vs. 0.32), executive functioning (*g* = 0.62 vs. −0.01), and speed of information processing (*g* = 0.33 vs. 0.06). These findings are surprising, given that effect sizes are usually larger in studies with inactive controls than in those with active controls (Cuijpers et al., 2024). It is possible that there was a higher dropout rate in the inactive control condition, as participants knew that they were not receiving treatment, which may have contributed to smaller effects by introducing attrition bias, whereby patients with lower baseline functioning—who may benefit the most from treatment—disproportionately dropped out from the control group. Active control trials are more likely to be real-world or later stage and may have higher baseline severity, which can lead to greater room for improvement, resulting in larger pre- to post changes for these individuals.

Aim 3d examined whether the intervention format (individual versus group) impacted treatment effects. There was no significant difference between individual and group interventions when examining pooled treatment effects. However, the point estimate of the effect size for the group format (*g* = 0.50) was qualitatively larger than for the individual format (*g* = 0.31). The results of the analysis examining cognitive outcome and format as moderators approached significance (*p* = 0.09). The point estimates of the treatment effects were larger for group formats relative to individual for the following cognitive outcomes: mental status and cognition (*g* = 0.58 vs. 0.16), memory (*g* = 0.57 vs. 0.37), executive functioning (*g* = 0.46 vs. 0.17). This finding indicates that group formats may be better for select cognitive outcomes for individuals with MCI. Past research examining the effects of group versus individual interventions has yielded mixed findings, with some research evaluating psychological therapies finding no difference (McRoberts et al., 1998), while others have shown greater benefits for individual formats (Cuijpers et al., 2008; Huntley et al., 2012). Similarly, mixed outcomes have been found in patient populations with dementia (Woods et al., 2012), and one study found that group settings were particularly effective at improving memory outcomes for midlife and older adults in residential care settings (Haslam et al., 2010). This may be in line with the findings of the current meta-analysis, suggesting that the social aspect of group interventions can play an important role in health and well-being in midlife and older adults, including those with MCI.

There are several limitations to this meta-analysis. First, several sub-group analyses had small and unbalanced group sizes, which may have reduced the statistical power to detect true differences between them. Second, even within subgroups, there was a high degree of heterogeneity across studies (i.e., different types of interventions used), making it difficult to isolate independent effects of specific strategies. There were also not enough instances of some variable levels of interest to justify including them in analysis. For example, only a single study evaluated a purely external strategy approach, so we were unable to evaluate the effectiveness of external strategies alone. Future research is needed to specifically test each of these strategy types or evaluate their effects in isolation. Finally, diagnostic assessments revealed significant evidence of small-study effects and publication bias, where smaller studies (assumed to have less precision) reported larger effect sizes. Although a sensitivity analysis demonstrated that the pooled treatment effect was still statistically significant after excluding studies with large standard errors, the effect size did drop from *g* = 0.37 to *g* = 0.22, implying that the true effect size may be lower (i.e., in the small range) than suggested in the main analysis.

This meta-analysis evaluated RCTs measuring the effectiveness of cognitive interventions with a compensatory component for improving cognitive outcomes in patients with MCI. Results showed that treatment effects were in the small-to-moderate range across cognitive outcomes. The interventions in the included studies had the largest effects on memory and working memory/attention outcomes. Interestingly, there was some evidence that, when compared to primarily compensatory approaches, multicomponent interventions (i.e., those that were partly compensatory but more heavily restorative) were better for improving certain cognitive processes, including working memory/attention, executive functioning, and speed of information processing. In some cases, there was also evidence that external strategies were more effective than internal strategies and that interventions were more effective when delivered in a group setting than when delivered individually.

## Supplementary Material

Supplementary Files

This is a list of supplementary files associated with this preprint. Click to download.

- MCIMetaAnalysisSupplement.docx

## Figures and Tables

**Figure 1.**
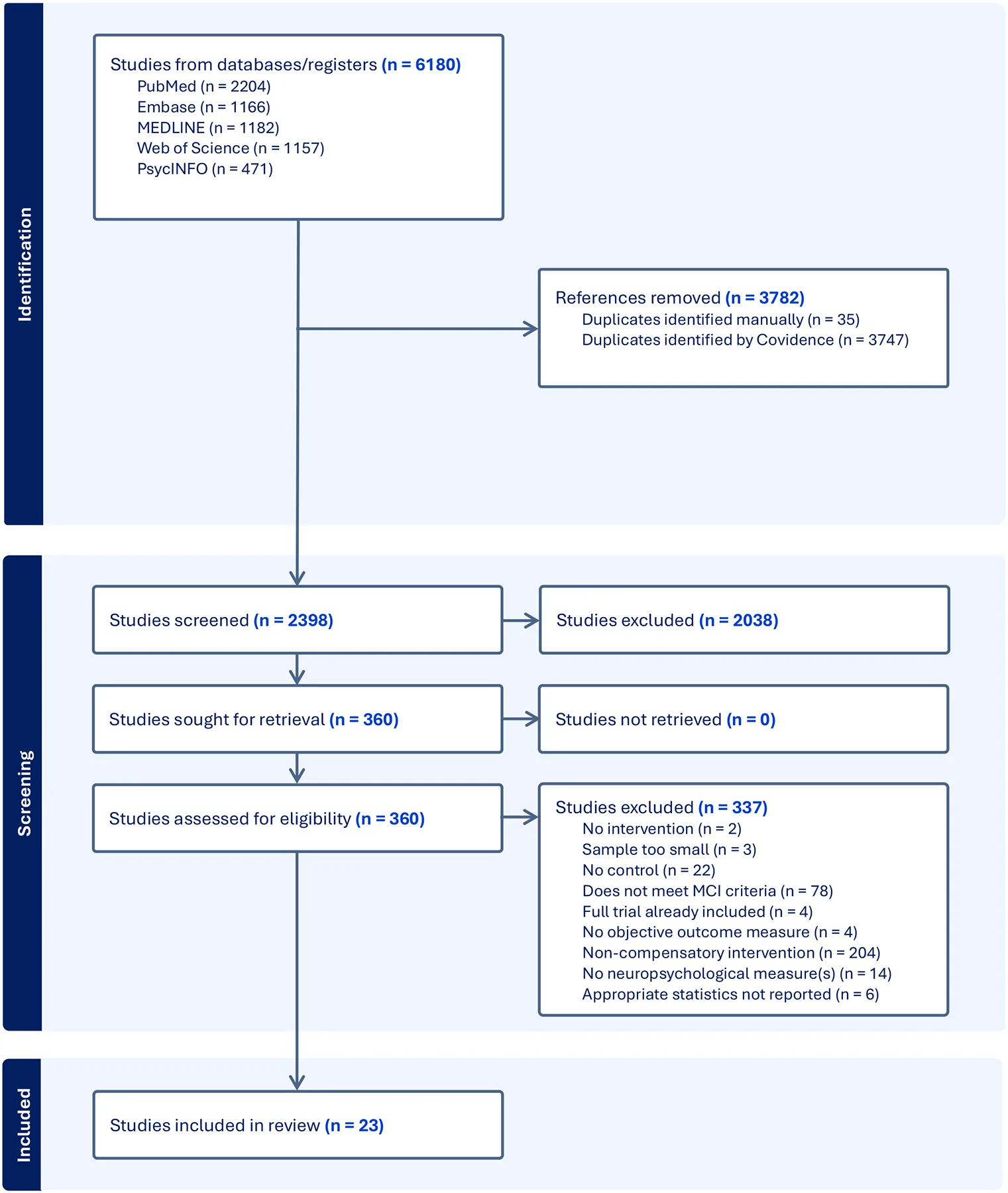
Meta-analysis flow diagram of reviewed literature.

**Figure 2.**
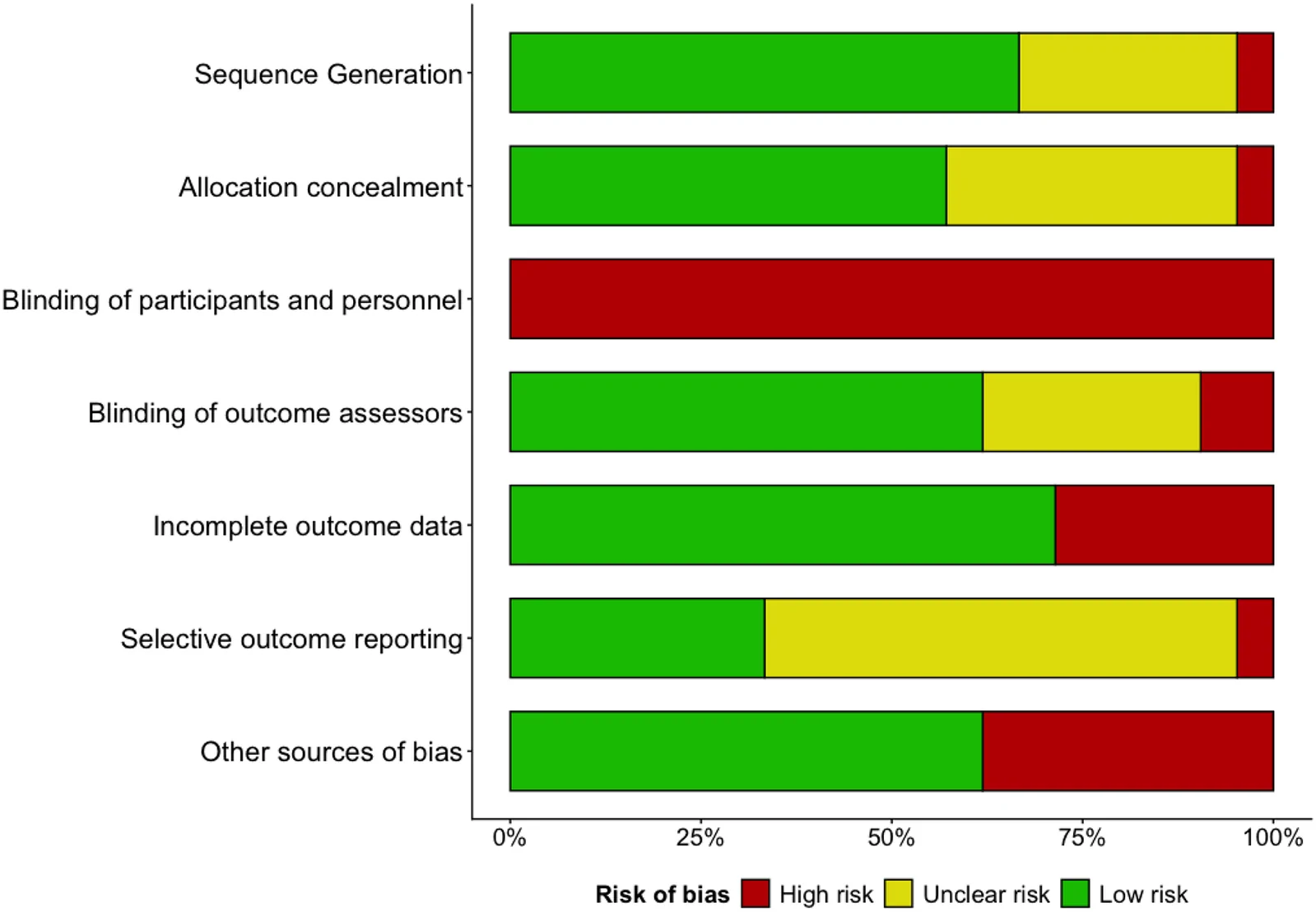
Risk of bias plot for included RCTs across the seven domains assessed.

**Figure 3.**
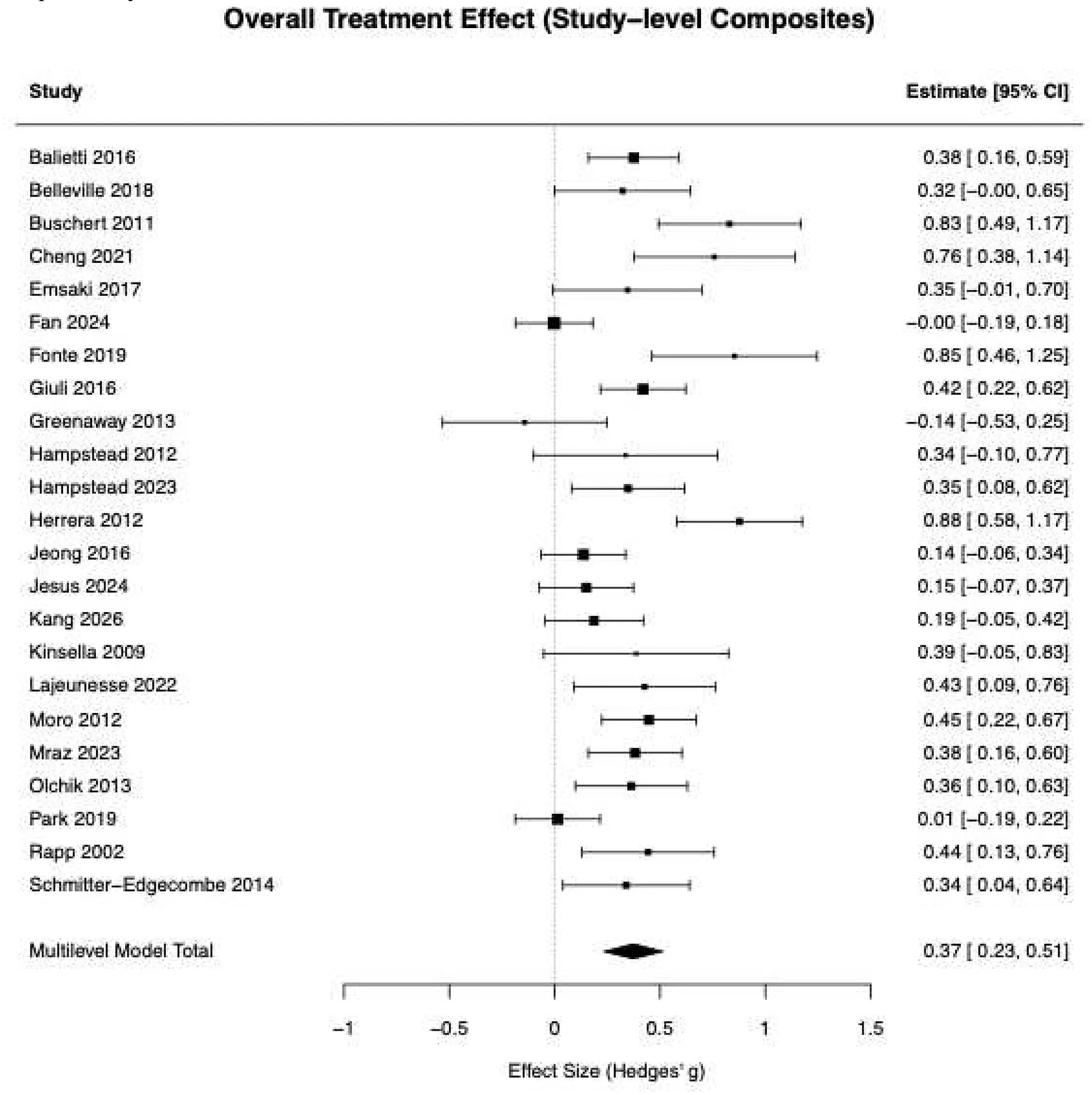
Aim 1: Meta-analysis of overall treatment effects for each study collapsing across different outcome domains.

**Figure 4.**
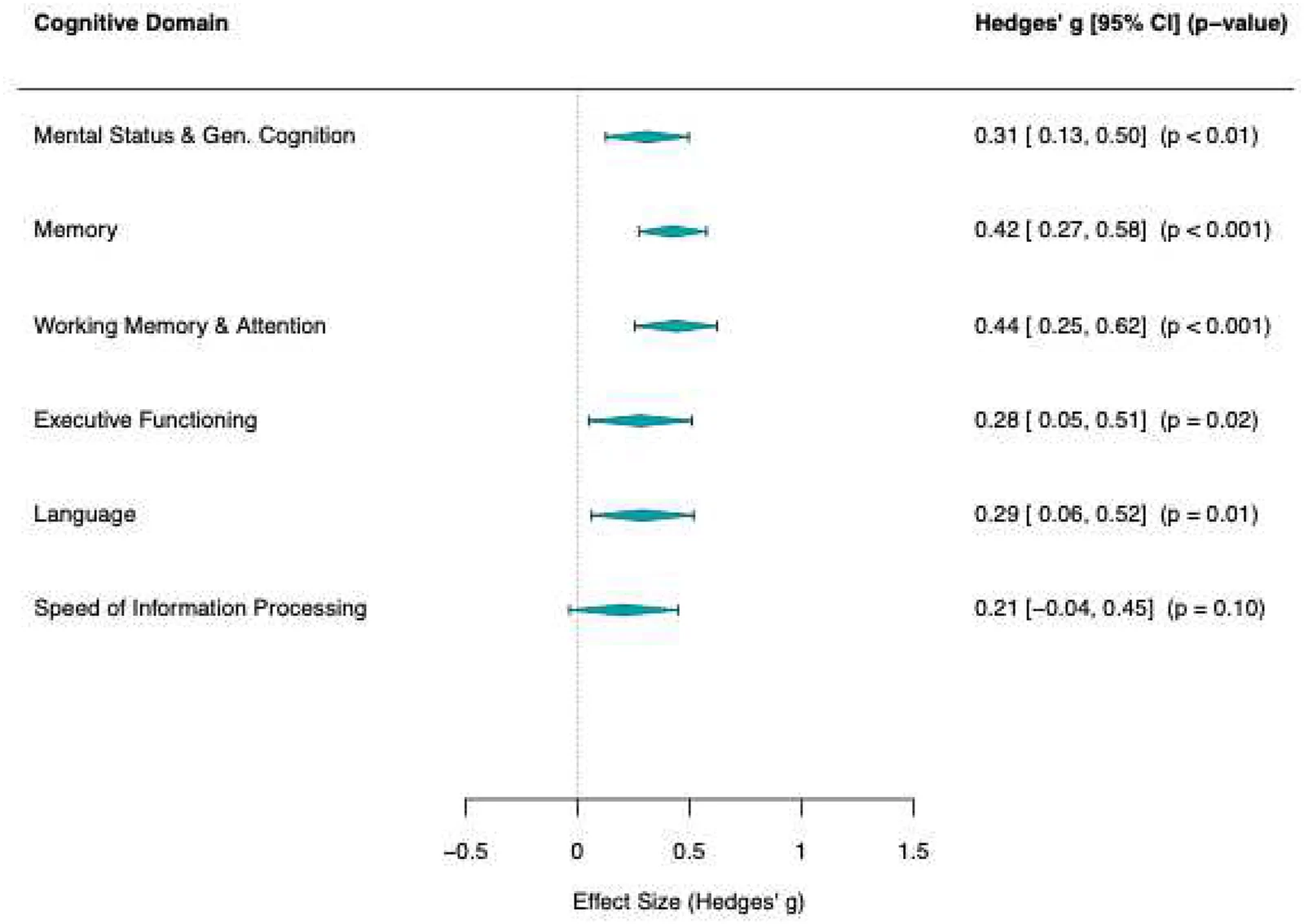
Aim 2: Meta-analysis of overall treatment effects by cognitive domain of outcome.

**Figure 5.**
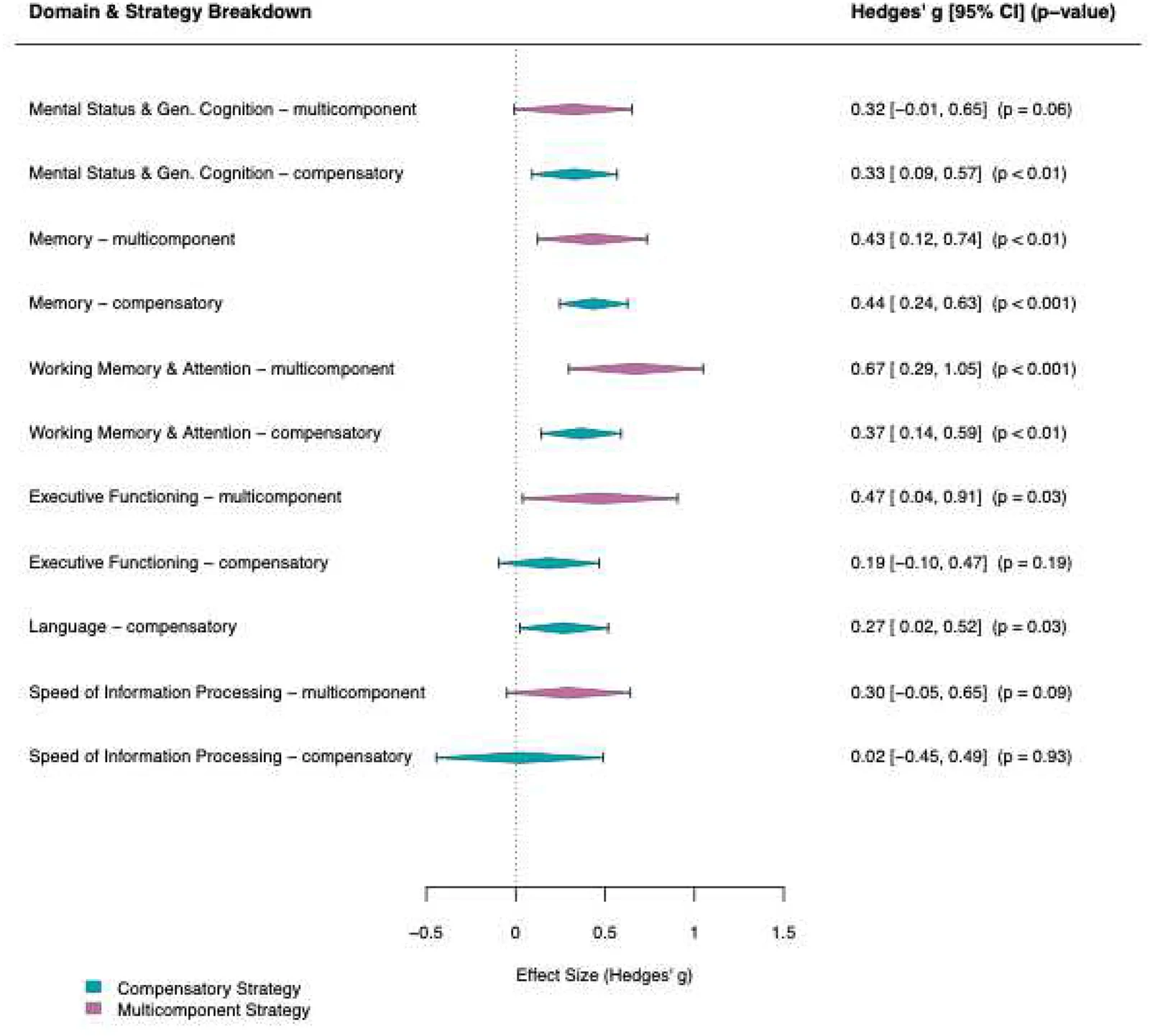
Aim 3a sub-group analyses: Meta-analysis of overall treatment effects by intervention type (compensatory vs. multicomponent) and cognitive domain of outcome.

**Figure 6.**
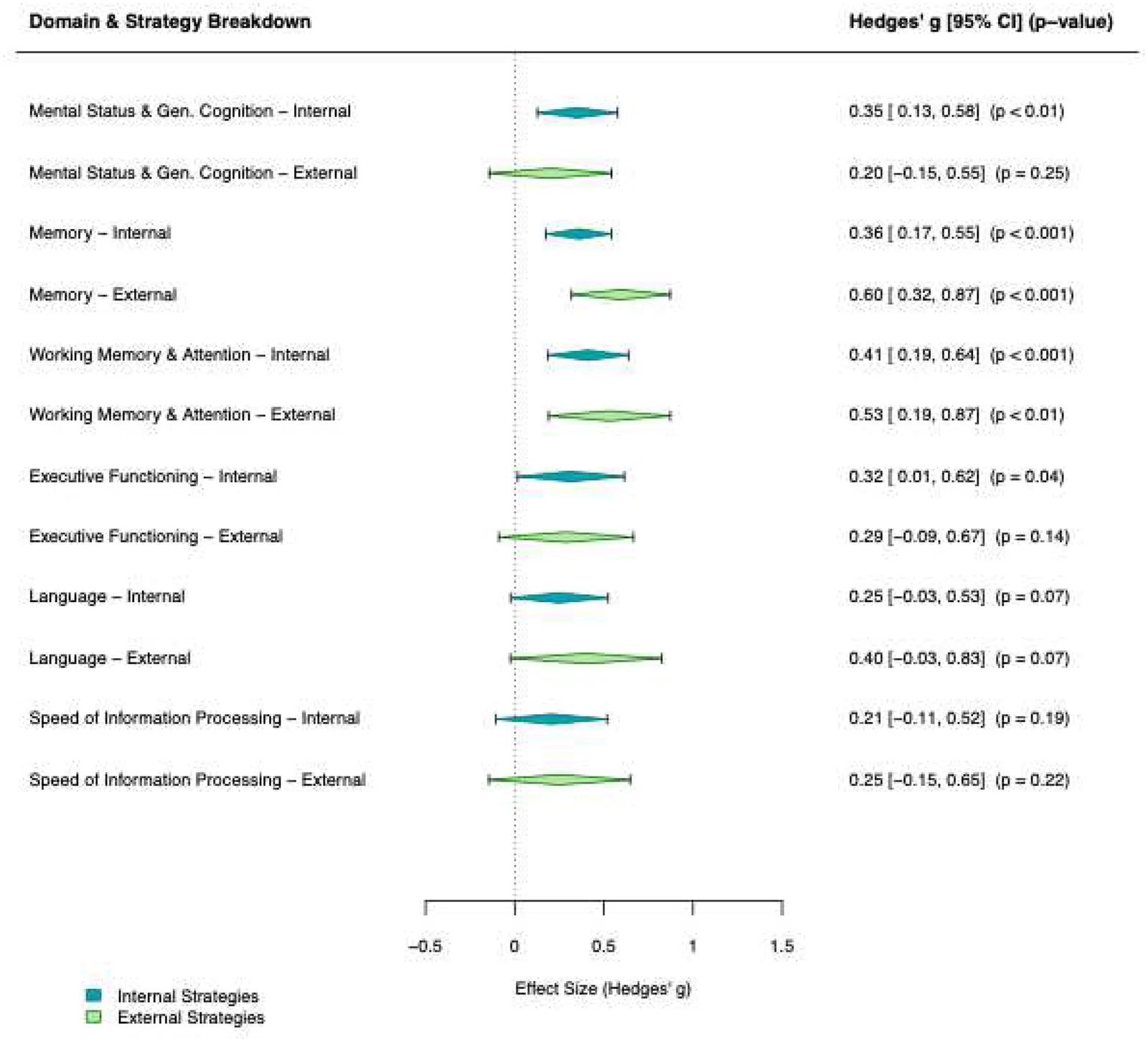
Aim 3b sub-group analyses: Meta-analysis of overall treatment effects by compensatory strategy type (internal vs. external) and cognitive domain of outcome.

**Figure 7.**
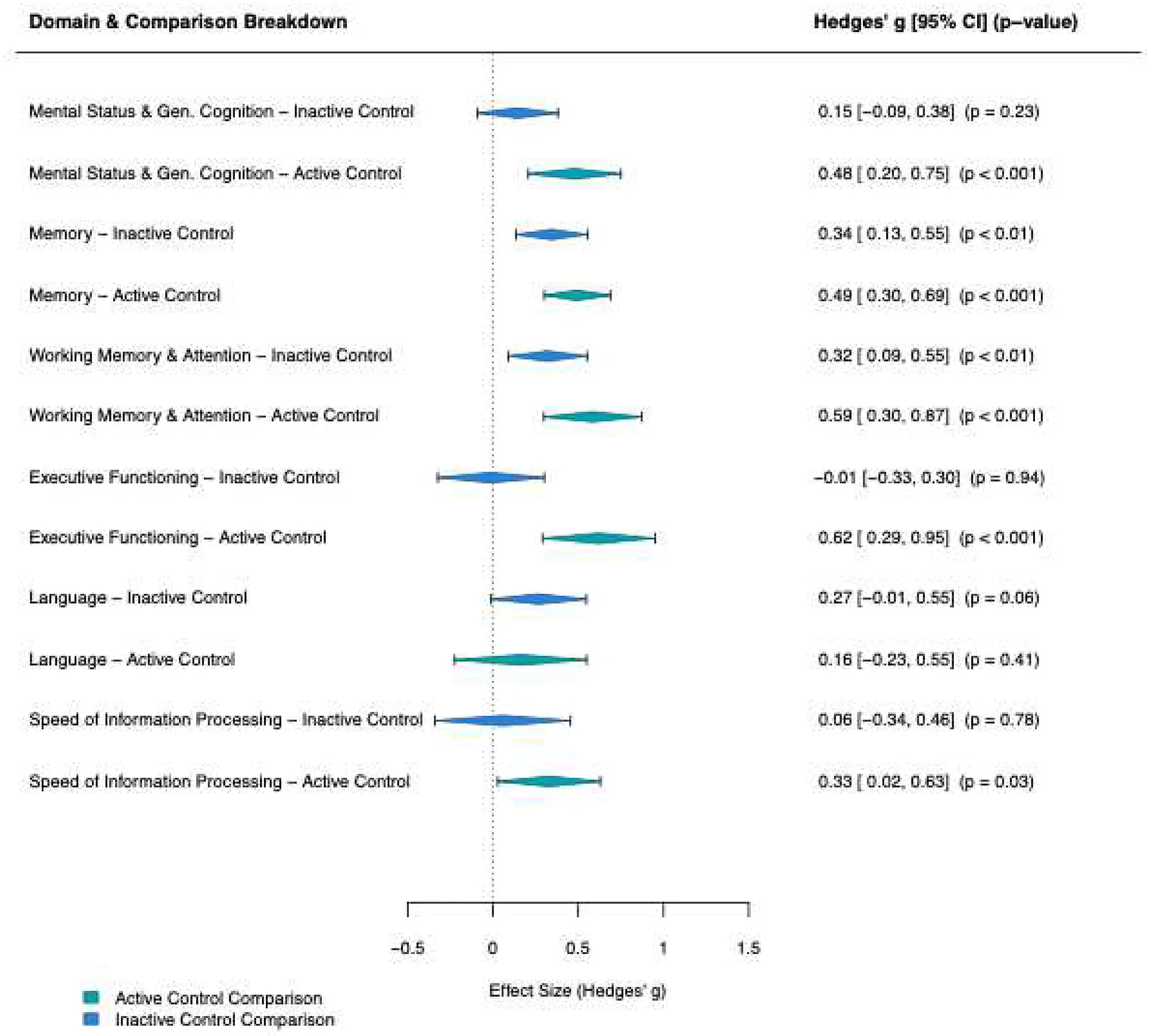
Aim 3c sub-group analyses: Meta-analysis of overall treatment effects by control group type (active vs. inactive) and cognitive domain of outcome.

**Figure 8.**
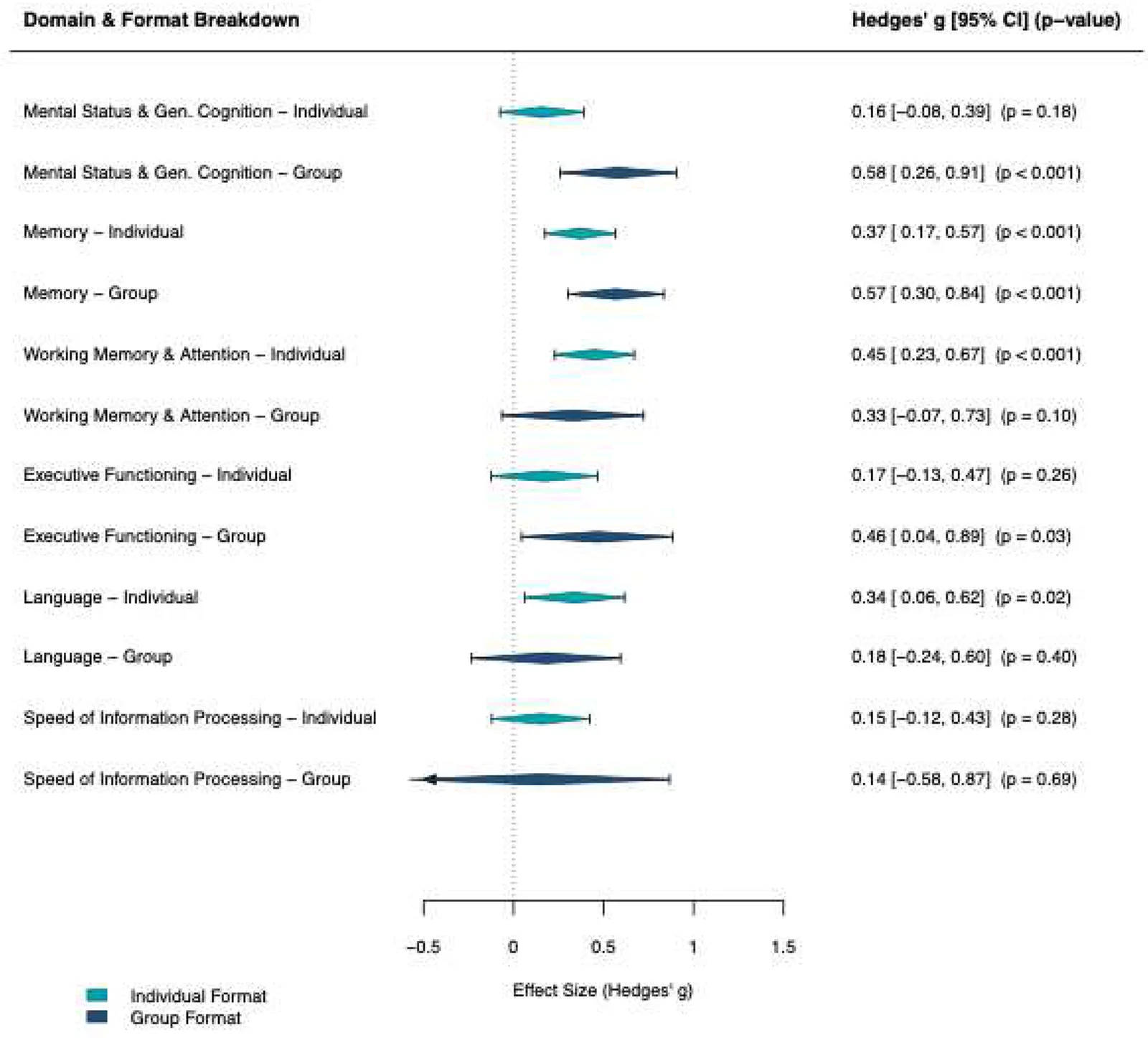
Aim 3d sub-group analyses: Meta-analysis of overall treatment effects by intervention format (individual, group, both) and cognitive domain of outcome.

**Table 1.** Compensatory strategies.

Strategy	Description
**External Strategies**	
Memory Aids	Calendars, notebooks, note systems, and prompts (Greenaway et al., 2013; Sohlberg et al., 2007; Wilson, 1999)
Cueing	Cue-based reminders prompt retrieval of information to facilitate recall performance (Belleville et al., 2006; Rogers & Milkman, 2016)
**Internal Strategies**	
Semantic Organization	Grouping information into semantic categories and by order of importance (Belleville et al., 2006; Hunt & McDaniel, 1993; Ley et al., 1973)
Chunking	Grouping information into smaller units or ‘chunks’ to reduce the load on working memory (Belleville et al., 2006; Miller, 1956; Thalmann et al., 2019)
Mental Imagery	The process of creating images in the mind, associating verbal and visual information to facilitate encoding and recall of information (Breuil et al., 1994; Paivio, 1969; Richardson, 1995)
Method of Loci	Items to be remembered are associated with specific locations within a mentally visualized environment to improve recall (Legge et al., 2012; Londos et al., 2008)

**Table 2.** Summary of study characteristics

Author	Study Design	Sample Size	Mean Age (SD)	Mean Mental Status Baseline (SD)	Format	Length	Intervention Components	Intervention category	Outcome Measures
Balietti (2016)	RCT, Inactive Control	Tx = 37 Ctrl = 33	Tx = 75.1 (1.0) Ctrl = 76.2 (1.0)	MMSE Tx = 24.5 (0.2) Ctrl = 27.2 (0.2)	Individual	Tx: 10 sessions, 1hr per session, 1x per week	Cognitive exercises, effective aids, and mnemonic strategies (errorless learning, spaced retrieval, organization, categorization, method of loci, visual imagery, face-name associations)	Compensatory, Internal	Memory: Word Pairing Learning Test, Prose Recall Test, Immediate Prose Recall, Delayed Prose Recall Working Memory & Attention: AMT, DS-FWD, CSST Language: SVF, PVF
Belleville (2018)	RCT, Active, Inactive Control	Tx = 49 Active Ctrl = 49 Inactive Ctrl = 47	Tx = 71.3 (8.5) Active Ctrl = 72.1 (6.7) Inactive Ctrl = 73.1 (6.5)	MoCA Tx = 24.1 (3.0) Active Ctrl = 25.0 (2.7) Inactive Ctrl = 24.2 (3.3)	Group	Tx: 8 sessions, 120-minute sessions, 1x per week Active Ctrl: 8 sessions, 120-minute sessions, 1x per week	Psychoeducational information, varying attentional priority, visual imagery, method of loci, name learning strategies, organization, mnemonic strategies	Compensatory, Internal	Memory: Immediate and Delayed episodic memory composite score (pooling z-scores of the word list and face-name association tasks)
Buschert (2011)	RCT, Active Control	Tx=12 Ctrl = 12	Tx = 71.8 (8.6) Ctrl = 70.7 (5.7)	MMSE Tx = 28.1 (1.5) Ctrl = 26.8 (1.5)	Group	Tx: 20 sessions, 120-minper session, 1x per week Active Ctrl: 6 sessions, 60-min per session, 1x per month	Cognitive exercises (e.g., attention, perception, and memory), restorative and compensatory mnemonic techniques (e.g., visual imagery, face-name association, use of external memory aids such as calendar, notes and prompts), errorless learning, and education	Multicomponent. Internal and External	Mental Status & General Cognition: ADAS-cog, MMSE Memory: RBANS-story memory, RBANS-story recall Executive Functioning: TMT-B Speed of Information Processing: TMT-A
Cheng (2021)	RCT, Active Control	Tx = 37 Ctrl = 33	Overall = 65.0 (6.5)	MMSE Overall = 27.00 (2.24)	Individual	Tx: Single session Active Ctrl: Single session	Mental Imagery	Compensatory, Internal	Memory: Plan Retention Index Executive Functioning: Plan Elaborateness Index, Plan Fidelity Index
Emsaki (2017)	RCT, Inactive Control	Tx=10 Ctrl = 10	Tx = 63.1 (5.6) Ctrl = 63.7 (7.1)	Not Reported	Individual	Tx: 5 sessions, 80-min per session, 1x per week	Memory specificity training: reduce over-generalized memories by increasing more specific memories	Compensatory, Internal	Memory: The Autobiographical Memory Test, WMS-visual immediate, WMS-visual delayed, WMS-auditory immediate, WMS-auditory delayed Working Memory & Attention: WMS-working memory
Fan (2024)	RCT, Active Control	Tx = 60 Ctrl = 60	Tx = 66.3 (4.4) Ctrl = 67.8 (5.1)	MMSE Tx = 28.4 (1.6) Ctrl = 29.1 (1.0)	Individual	Tx: 6-weeks in-person sessions (6 sessions, 90-min, 1x per week), 6-weeks computerized training (18 sessions, 30-min, 3x per week), daily homework (30 min/day) Active Ctrl: 12 sessions, 30-min, 1x per week	Memory training (picture recall, categorization, cueing, repetition, association, sentence formation, story construction, utilization of the Roman room method, face-name association, and verbalization to aid retention), attention-related training (character-picture association, idiom-picture association, and spot-the-difference tasks were incorporated), lifestyle, computerized Interaction	Multicomponent, Internal	Mental Status & General Cognition: MMSE, MoCA Memory: LMT-immediate recall, LMT-delayed recall, AVLT-immediate memory, AVLT-delayed recall Working Memory & Attention: DS-FWD, DS-BWD, DS Executive Functioning: DSST-wrong number Speed of Information Processing: DSST, TMT-A
Fonte (2019)	RCT, Active Control	Tx=11 Ctrl = 9	Tx = 76 (5) Ctrl = 79 (3)	MMSE Tx = 26.4 (1.4) Ctrl = 25.7 (1.8)	Group	Tx: 72 sessions, 90 min sessions, x3 per week Active Ctrl: standard pharmacological treatment	Visual imagery, fece-name associations, external aids (calendars, notes, and prompts), ecological daily-life tasks	Compensatory, Internal and External	Memory: RBMT Executive Functioning: FAB, TMT-B Speed of Information Processing: TMT-A
Giuli (2016)	RCT, Inactive Control	Tx = 48 Ctrl = 49	Tx = 76.0 (6.3) Ctrl = 76.5 (5.7)	MMSE Tx = 25.7 (1.8) Ctrl = 25.8 (1.9)	Individual	Tx: 10 sessions, 45-min per session, 1x per week	Strategies for orientation, memory, categorization, clusterning, compensation strategies, aids, psychological support, education on healthy lifestyles, at home cognitive exercises.	Compensatory, Internal and External	Mental Status and General Cognition: MMSE Memory: Prose Memory Test, Word Pairing Learning Test Working Memory & Attention: DS-FWD, DS-BWD, AMT, CSST Language: SVF, PVF
Greenaway (2013)	RCT, Inactive Control	Tx = 20 Ctrl = 20	Tx = 72.7 (6.9) Ctrl = 72.3 (7.9)	MMSE Tx = 26.4 (2.2) Ctrl = 27.2 (2.4)	Individual	Tx: 12 sessions, 60-min per session, 2x per week	Memory Support System (MSS): training in the use of a calendar and note-taking system	Compensatory, External	Mental Status and General Cognition: MMSE, DRS-2
Hampstead (2012)	RCT, Active Control	Tx = 20 Ctrl = 20	Tx = 73.5 (10.1) Ctrl = 70.5 (5.8)	MMSE Tx = 26.9 (1.8) Ctrl = 26.1 (2.1)	Individual	Tx: 3 sessions in two-week period, 60–90 min Active Ctrl: matched exposure	Mnemonic strategy training	Compensatory, Internal	Memory: Accuracy-trained list, Accuracy-untrained list
Hampstead (2023)	RCT, Active Control	Tx = 28 Ctrl = 29	Tx = 72.7 (8.3) Ctrl = 70.5 (8.3)	MMSE Tx = 27.2 (1.5) Ctrl = 27.8 (2.2)	Individual	Tx: 3 sessions in two-week period Active Ctrl: Spaced retrieval training	Mnemonic strategy training	Compensatory, Internal	Memory: Accuracy-trained list, Accuracy-untrained list, OLTT-free recall, OLTT-cued recall, OLTT-recognition
Herrera (2012)	RCT, Active Control	Tx=11 Ctrl = 11	Tx = 75.1 (2.0) Ctrl = 78.2 (1.4)	MMSE Tx = 27.4 (0.5) Ctrl = 27.2 (0.4)	Individual	Tx: 24 sessions, 60-min per session, 2x per week Active Ctrl: 24 sessions, 60-min per session, 2x per week	Cognitive training targeting memory and attention (e.g., visual recognition, working memory, focused and divided attention tasks) and mnemonic strategies (naming, repetition, categorization, and association)	Multicomponent, Internal	Memory: 16-FR/CR, MMSE-recall, DMS48, DPT-recognition subtest A & B, RCFT, BEM-144 Working Memory & Attention: DS-FWD, DS-BWD
Jeong (2016)	RCT, Inactive Control	Tx = 96 Ctrl = 99	Tx = 70.8 (6.9) Ctrl = 71.6 (6.5)	MMSE Tx = 25.9 (2.5) Ctrl = 25.3 (2.5)	Group	Tx: 24 sessions, 90-min per session, 2x per week	Memory strategies: categorization, story making, visual imagery, association, spaced retrieval, saying something out loud to remember it, hierarchical organization, errorless learning, finding key words, face-name association, cueing, repetition, and practice filling in the blanks	Compensatory, Internal	Mental Status & General Cognition: MMSE, CDR-SB, Modified ADS-cog Memory: LMT, PMT Working Memory & Attention: Working Memory Composite Score (average z-scores from DS-FWD and DS-BWD) Executive Functioning: Executive functioning Composite Score (average z-scores from Stroop, COWAT, Animal Fluency, DSST)
Jesus (2024)	RCT, Active Control	Tx = 99 Ctrl = 101	Tx = 79.1 (5.4) Ctrl = 80.3 (4.7)	ACE-R Tx = 66.4 (9.1) Ctrl = 67.6 (9.2)	Individual	Tx: 10 sessions, 90-min per session, 1x per week Active Ctrl: usual care	Cognitive Training (selective and sustained attention training, episodic memory training), Psychotherapeutic Intervention (psychoeducation, cognitive restructuring, behavioral activation, problem-solving skills), compensatory strategies (external and internal e.g., calendars, association, organization)	Multicomponent, Internal and External	Mental Status & General Cognition: ACE-R Memory: LMT-immediate recall, LMT-delayed recall Speed of Information Processing: DSST
Kang (2026)	RCT, Inactive Control	Tx = 50 Ctrl = 150	Tx = 75.0 (5.8) Ctrl = 74.8 (5.8)	MMSE Tx = 23.9 (2.8) Ctrl = 23.0 (2.7)	Individual	Tx: 168 sessions, 15-min per session, 2x per day for 12 weeks	Attention training, Imagination training (visual imagery), Association training.	Multicomponent, Internal	Mental Status & General Cognition: ADAS-cog, MMSE, CDR-SB, ADCOMS Speed of Information Processing: DSST
Kinsella (2009)	RCT, Inactive Control	Tx = 26 Ctrl = 28	Tx = 76.9 (5.7) Ctrl = 74.7 (6.1)	MMSE Tx = 25.9 (2.8) Ctrl = 26.8 (1.8)	Group	Tx: 5 sessions, 90-min per session, 1x per week	Memory education, training in use of external memory aids, improving organizational and attention skills, categorization, visual imagery, errorless learning, spaced retrieval, coping strategies	Compensatory, Internal and External	Memory: Prospective Memory Performance
Lajeunesse (2022)	RCT, Inactive Control	Tx=12 Ctrl = 12	Tx = 73.8 (7.3) Ctrl = 76.3 (5.4)	MoCA Tx = 25.4 (2.8) Ctrl = 24.5 (2.6)	Individual	Tx: 10 sessions, 60–120-mins per session, 1x per week	Visual Imagery training program to help patients create mental pictures to strengthen the cue-action association to facilitate subsequent retrieval	Compensatory, Internal	Mental Status & General Cognition: MoCA Memory: TEMP, Retrospective Memory Composite Score (average z-scores from AVLT-immediate recall, AVLT-delayed recall, AVLT-delayed recognition, LMT-immediate recall, LMT-delayed recall) Working Memory & Attention: Attention/working memory composite score (average z-scores from DS, Symbol Cancellation Test, DS-FWD, DS-BWD, Color-Word Inference Test) Executive Functioning: Executive Functioning Composite Score (average z-scores from TMT-A, TMT-B, Color-Word Interference Test, Verbal Fluency Test, Mazes)
Moro (2012)	Controlled, Inactive Control	Tx=15 Ctrl = 15	Tx = 73.3 (6.9) Ctrl = 68.5 (8.7)	Not Reported	Individual	Tx: Month 1:3 sessions per week; Months 2–6:1 session per week	Strategies such as verbal and visual association, organization, categorization, visualization, anticipation and retrospection, mental imagery, and external aids	Compensatory, Internal and External	Memory: AVLT-immediate recall, AVLT-delayed recall, AVLT-omissions, AVLT-false recognitions, Story Recall Working Memory & Attention: Bell test, AMT, Bourdon Test, Verbal Span, Listening Span Test Executive Functioning: Tower of London, Test of Analogies, Stroop, TMT-B-A Language: VFT Speed of Information Processing: TMT-A
Mraz (2023)	Controlled, Active Control	Tx = 20 Ctrl = 19	Tx = 80.9 (5.9) Ctrl = 78.4 (9.5)	MMSE Tx = 23.7 (1.8) Ctrl = 23.6 (1.7)	Group	Tx: 10 sessions, 90-mins per session, 1x per week Active Ctrl: 10 sessions, 90-mins per session, 1x per week	Trained in the use of chunking association method and honeycomb concept and implemented this encoding strategy in different tasks each week	Compensatory, Internal	Mental Status & General Cognition: Cognistat (orientation, construction, calculation, similarity, judgement subtests) Memory: Cognistat-memory, CERAD-word list memory, CERAD-word list recall Working Memory & Attention: Cognistat (attention subtest) DS-FWD, DS-BWD Language: Cognistat (comprehension, repetition, naming subtests)
Olchik (2013)	RCT, Active, Inactive Control	Tx=16 Active Ctrl = 17 Inactive Ctrl = 14	Tx = 70.3 (4.3) Active Ctrl = 72.2 (6.3) Inactive Ctrl = 70.2 (5.7)	MMSE Tx = 28.3 (0.9) Active Ctrl = 27.9 (1.9) Inactive Ctrl = 28.2 (1.7)	Group	Tx: 8 sessions, 90-mins, 2x per week Active Ctrl: 8 sessions, 90-mins, 2x per week	Education on memory and aging, memory strategies: active attention, categorization, association, and visual imagery, exercises to practice strategies	Compensatory, Internal	Memory: AVLT-learning score, AVLT-immediate recall, AVLT-delayed recall, RBMT-screening score, RBMT-immediate story, RBMT-delayed story Language: Categorical Verbal Fluency, FAS Verbal Fluency
Park (2019)	RCT, Inactive Control	Tx = 31 Ctrl = 26	Tx = 70.7 (7.5) Ctrl = 69.7 (8.4)	MMSE Tx = 25.5 (2.3) Ctrl = 25.9 (2.8)	Individual	Tx: 30 sessions, 30-minper session, 3x per week	Memory training: time difference, error exclusion learning, categorization, three-phasic method, face recognition, imagery, exercise, and iterative methods; Frontal training: behavior modification, ordering, abstract thinking training, and goal-directed training. Attention training: attention processing, Sudoku, color-diagram finding, shape diagram finding, and word finding; Visuospatial function training: direction training and localization training; Language training: word naming test, country naming test, memory, writing, and speaking events; Orientation training: real sense training and drawing a clock and calculation training	Compensatory, Internal	Mental Status & General Cognition: ADAS-cog, MMSE, CDR-SB Memory: story memory (immediate recall, delayed recall, recognition, total score) Working Memory & Attention: DS-FWD, DS-BWD, DS-total Executive functioning: COWAT-category fluency, COWAT-letter, COWAT-total, Stroop Speed of Information Processing: DSST
Rapp (2002)	RCT, Inactive Control	Tx = 9 Ctrl = 10	Tx = 73.3 (6.6) Ctrl = 75.1 (7.0)	MMSE Tx = 28.0 (1.5) Ctrl = 27.3 (1.9)	Group	Tx: 6 sessions, 120-mins, lx per week	Multifaceted training intervention; includes education on memory, relaxation skills training, mnemonic strategies, and cognitive restructuring to change beliefs about memory control.	Compensatory, Internal	Memory: Word list (immediate, delayed), shopping list (immediate, delayed), names and faces (immediate, delayed), paragraph (immediate, delayed)
Schmitter-Edgecombe (2014)	RCT, Active Control	Tx = 23 Ctrl = 23	Tx = 73.0 (7.1) Ctrl = 73.3 (7.9)	RBANS Tx = 82.0 (11.5) Ctrl = 86.7 (15.2)	Individual and Group	Tx: 2 individual sessions + 20 group sessions, 120-mins, 2x per week Active Ctrl: usual care	Educational workshops, memory strategy training (to-do-lists, calendars, internal memory strategies), problem solving	Multicomponent, Internal and External	Memory: RBMT, RBANS-immediate memory, KBAJNS-delayed memory Executive Functioning: EFPT-bill paying

*Note*. **RCT** = Randomized Controlled Trial; **Tx** = Treatment Intervention; **Ctrl** = Control. Measure Acronyms: **16-FR/CR** = 16-item Free and Cued Reminding Test; **ACE-R** = Addenbrooke’s Cognitive Examination–Revised; **ADAS-cog** = Alzheimer’s Disease Assessment Scale–cognitive subscale; **ADCOMS** = Alzheimer’s Disease Composite Score; **AMT** = Attentive Matrices Test; **AVLT** = Auditory Verbal Learning Test; **BEM-144** = Memory Battery (144 items); **CDR-SB** = Clinical Dementia Rating Scale–Sum of Boxes; **CERAD** = Consortium to Establish a Registry for Alzheimer’s Disease; **Cognistat** = Neurobehavioral Cognitive Status Examination; **COWAT** = Controlled Oral Word Association Test; **CSST** = Corsi Supraspan Test; **DMS48** = Delayed Matching-to-Sample Task (48 items); **DPT** = Doors and People Test; **DRS-2** = Mattis Dementia Rating Scale–2; **DS** = Digit Span Test; **DS-BWD** = Digit Span Backward; **DS-FWD** = Digit Span Forward; **DSST** = Digit Symbol Substitution Test; **EFPT** = Executive Function Performance Test; **FAB** = Frontal Assessment Battery; **LMT** = Logical Memory Test; **MMSE** = Mini-Mental State Examination; **MoCA** = Montreal Cognitive Assessment; **OLTT** = Object-Location Touchscreen Test; **PMT** = Prospective Memory Test; **PVF** = Phonemic Verbal Fluency Test; **RBANS** = Repeatable Battery for the Assessment of Neuropsychological Status; **RBMT** = Rivermead Behavioral Memory Test; **RCFT** = Rey Complex Figure Test; **SVF** = Semantic Verbal Fluency Test; **TEMP** = Ecological Test of Prospective Memory; **TMT** = Trail Making Test; **VFT** = Verbal Fluency Test; **WMS** = Wechsler Memory Scale.

## Data Availability

The data that support the findings of this study are available from the corresponding author, AH, upon reasonable request.
